# β-Catenin Regulates Cardiac Energy Metabolism in Sedentary and Trained Mice

**DOI:** 10.3390/life10120357

**Published:** 2020-12-17

**Authors:** Volodymyr V. Balatskyi, Oksana L. Palchevska, Lina Bortnichuk, Ana-Maria Gan, Anna Myronova, Larysa L. Macewicz, Viktor O. Navrulin, Lesya V. Tumanovska, Adam Olichwier, Pawel Dobrzyn, Oksana O. Piven

**Affiliations:** 1 Department of Human Genetics, Institute of Molecular Biology and Genetics, National Academy of Sciences of Ukraine, 150 Akademika Zabolotnogo Street, 03680 Kyiv, Ukraine; v.balatskyi@nencki.edu.pl (V.V.B.); opalchevska@iimcb.gov.pl (O.L.P.); lina.bortnichuk@ukr.net (L.B.); myronova@rhrk.uni-kl.de (A.M.); l.l.macewicz@imbg.org.ua (L.L.M.); 2 Laboratory of Molecular Medical Biochemistry, Nencki Institute of Experimental Biology, Polish Academy of Sciences, 3 Pasteur Street, 02-093 Warsaw, Poland; a.gan@nencki.edu.pl (A.-M.G.); v.navrulin@nencki.edu.pl (V.O.N.); a.olichwier@nencki.edu.pl (A.O.); 3 Laboratory of Neurodegeneration, International Institute of Molecular and Cell Biology in Warsaw, 46-580 Warsaw, Poland; 4 Department of General and Molecular Pathophysiology, Bogomoletz Institute of Physiology, National Academy of Sciences of Ukraine, 4 Bogomoletz Street, 01024 Kyiv, Ukraine; ltumanovska@biph.kiev.ua

**Keywords:** Wnt/β-catenin signaling, training-induced heart hypertrophy, glucose metabolism, lipid metabolism, β-oxidation, oxidative phosphorylation

## Abstract

The role of canonical Wnt signaling in metabolic regulation and development of physiological cardiac hypertrophy remains largely unknown. To explore the function of β-catenin in the regulation of cardiac metabolism and physiological cardiac hypertrophy development, we used mice heterozygous for cardiac-specific *β-catenin* knockout that were subjected to a swimming training model. *β-Catenin* haploinsufficient mice subjected to endurance training displayed a decreased β-catenin transcriptional activity, attenuated cardiomyocytes hypertrophic growth, and enhanced activation of AMP-activated protein kinase (AMPK), phosphoinositide-3-kinase–Akt (Pi3K–Akt), and mitogen-activated protein kinase/extracellular signal-regulated kinases 1/2 (MAPK/Erk1/2) signaling pathways compared to trained wild type mice. We further observed an increased level of proteins involved in glucose aerobic metabolism and β-oxidation along with perturbed activity of mitochondrial oxidative phosphorylation complexes (OXPHOS) in trained *β-catenin* haploinsufficient mice. Taken together, Wnt/β-catenin signaling appears to govern metabolic regulatory programs, sustaining metabolic plasticity in adult hearts during the adaptation to endurance training.

## 1. Introduction

Endurance training causes the development of physiological heart hypertrophy, also known as athletic heart. Physiological heart remodeling is associated with enhanced heart function, increased heart mass, and coronary blood flow [1,2,3]. Physiological hypertrophy is also characterized by increased glucose and fatty acids (FA) oxidation. Furthermore, endurance training stimulates mitochondrial biogenesis in various animal models [4]. Pathological heart hypertrophy (e.g., caused by hypertension), in turn, is accompanied by a reduction of FA uptake and oxidation, mitochondrial dysfunction, and triacylglycerols (TAG) accumulation [4,5]. Pressure-induced pathological cardiac hypertrophy is orchestrated by calcineurin–nuclear factor of activated T cells pathway, Janus kinase–signal transducers, and activators of transcription pathway, Pi3K–Akt, MAPK/Erk1/2, canonical Wnt, and G-protein-coupled receptor signaling cascades [2,3], whereas physiological hypertrophy is accompanied by increased Pi3K–Akt and AMPK signaling activities [5]. Both physiological and pathological hypertrophy are characterized by increased cardiomyocytes size that is governed by common Pi3K–Akt and MAPK/Erk1/2 signaling pathways [6,7] which have crosstalks with β-catenin signaling [8]. Thus, it is tempting to suggest that β-catenin can also be involved in the development of physiological hypertrophy.

Canonical Wnt signaling and β-catenin play central roles in both heart development and normal heart homeostasis [9,10,11,12,13,14,15]. While activation of β-catenin signaling is known to direct the development of maladaptive heart remodeling in response to pathological stimuli [9,10,11,12,13,14,15,16], the involvement of canonical Wnt signaling in training-induced heart remodeling has not yet been studied. However, there is fragmentary evidence suggesting the involvement of β-catenin in the response to physical activity. For example, it has been shown that long-term training decreases the levels of circulating Wnt inhibitors Dickkopf-related protein 1 and Secreted frizzled-related protein 1 in patients with breast cancer [17]. In addition, power training stimulates the activation of β-catenin in skeletal muscles of healthy volunteers [18].

Emerging evidence suggests that canonical Wnt signaling plays an essential role in the regulation of cellular metabolism, including FA metabolism and the regulation of a peroxisome proliferator-activated receptor α (PPARα) via c-Myc, a β-catenin target [19]. Moreover, canonical Wnt signaling may play a crucial role in regulating mitochondrial energy metabolism and mitochondrial homeostasis, since activation of canonical Wnt signaling in C2C12 myoblasts is reported to stimulate mitochondrial proliferation and mitochondrial oxidative function [20]. In addition, active β-catenin restores mitochondrial function in the brains of parkinsonian rats [21], but hepatocyte-specific overexpression of constitutively active β-catenin in mice leads to early lethality due to mitochondrial dysfunction [22]. Overactive β-catenin also shifts the metabolism of cancer cells from oxidative to anaerobic [23]. Thus, while the role of β-catenin in regulating mitochondrial function may be specific to certain tissue, it has yet to be studied in the heart.

Here we employed a tissue-specific knockout approach to explore the role of Wnt/β-catenin signaling in heart remodeling during the adaptation to endurance training. We demonstrated that canonical Wnt signaling plays an important role in the progression of physiological hypertrophic cardiomyocytes growth. The cardiospecific *β-catenin* knockout attenuates hypertrophic cardiomyocytes growth, upregulates fetal genes, decreases heart rate, and modulates heart energetic metabolism under physiological conditions and after the exercise. Downregulation of canonical Wnt affects the cardiomyocytes signaling machinery and activates the main regulators of hypertrophic remodeling, such as cyclic adenosine monophosphate-dependent protein kinase A (PKA), Akt, and MAPK/Erk1/2 kinases. In aggregate, our data imply the critical signaling function of β-catenin for heart remodeling during the adaptation to endurance training.

## 2. Materials and Methods

### 2.1. Mouse Breeding and Genotyping

To generate cardiac-specific deletion of *β-catenin (ctnnb1)*, α-myosin heavy chain (αMHC)-Cre mice were mated with homozygous floxed *β-catenin* mice as described by Piven et al. [24]. The αMHC-Cre mice were described previously [25]. This αMHC-Cre transgene elicited recombination in cardiac myocytes, but not in other cell types. *Ctnnb1*^floxp/floxp^ mice were obtained from Jackson Laboratories (Bar Harbor, ME, USA). For all experiments and analyses, the following genotypes were utilized: *Ctnnb1*^floxp/Wt^; αMHC-Cre^+^ (are referred to as WT/CKO) and *Ctnnb1*^floxp/floxp^; αMHC-Cre^−^; αMHC-Cre^−^ and *Ctnnb1*^floxp/Wt^; αMHC-Cre^−^ (are referred to as WT/WT). Tail tip tissues were used for genotyping. DNA isolation, polymerase chain reaction, and primers were described previously [26]. 

All animals were treated in accordance with Council Directive on the approximation of laws, regulations, and administrative provisions of the Member States regarding the protection of animals used for experimental and other scientific purposes (86/609/EEC), and the Declaration of Helsinki. The Institute of Molecular Biology and Genetics of National Academy of Science of Ukraine Ethical Committee has approved all the experimental procedures (Protocol Number 15/01.11.2018). 

### 2.2. Training Protocol

Three-months-old male mice were used for swimming training, which is known to induce stronger heart hypertrophy compared with running [27]. The protocol for the swimming test was adopted from Evangelista et al. [28]. The experimental animals were placed in a 40 × 60-cm-large, 20-cm-deep container containing warm water (25–30 °C) which required them to swim. The container was deep enough to prevent mice from clinging. Exercise duration started from five minutes and increased every next day for 90 s over the 38-day experimental period (five d/wk). When the period of swimming reached 60 min 30 s, mice where kept for one more day without training to measure heart rate in resting state. Animals were sacrificed by cervical dislocation, and all tissue samples were dissected and processed immediately or kept in liquid nitrogen until used.

### 2.3. Heart Rate and Heart Weight/Tibia Length Ratio Measurement

The next day after training, the mice were anesthetized with xylazine and thiopental [29,30]. Electrodes were inserted subcutaneously; two at the ventral junction between the chest and the right and left forelimbs, and the third at the junction between the lower abdomen and left hind limb. The electrodes were connected to a homemade electrocardiogram (ECG) amplifier. ECG was recorded using in-house software for at least 15 min and then averaged measurements of 100 successive cycles were used to determine the heart rate (WT/WT control, *n* = 5, WT/WT training, *n* = 4, WT/CKO control, *n* = 4, WT/CKO training *n* = 4). After the animals were sacrificed, the tibia length (TL) was measured. The heart was excised, washed with 1 M KCl in PBS and blotted dry, and then the heart weight (HW) was measured. These parameters were used to calculate the HW/TL ratio. WT/WT control, *n* = 11, WT/WT training *n* = 11, WT/CKO control, *n* = 7, WT/CKO training *n* = 12.

### 2.4. Histological Analysis

Hearts were fixed in 4% paraformaldehyde, dehydrated, and embedded in paraffin. General heart morphology was determined using 9 μm deparaffinized transverse hematoxylin and eosin (H&E) stained sections. The cardiomyocyte cross-section area was measured at the level of the nuclei in the left ventricle at the level of anterior papillary muscle. Over 100 cells per heart from 3 hearts of each genotype were analyzed. Tissue fibrosis was detected using van Gieson’s stain. Frozen sections of heart tissue were prepared as described elsewhere [31]. The cross-sectional heart area was analyzed using light microscopy Primo Star (Carl Zeiss, Gӧttingen, Germany). 

### 2.5. Oxidative Phosphorylation Histochemistry

Histochemical evaluation of the activity of NADH-coenzyme Q oxidoreductase (complex I), succinate dehydrogenase (complex II) and cytochrome c oxidase (complex IV) was performed as described elsewhere [32,33]. Briefly, frozen sections were maintained for one hour at room temperature, rinsed in 0.1 M PBS (pH 7.0), and stained in appropriate solution for 40 min at 37 °C: (1) complex I—(0.625 mg/mL NADH and 1.5 mM nitroblue tetrazolium in 0.1 M PBS, pH 7.0), (2) complex II—(1.5 mM nitroblue tetrazolium, 130 mM sodium succinate, 0.2 mM phenazine methosulfate and 1.0 mM sodium azide in 0.1 M PBS, pH 7.0), and (3) complex IV—(0.5 mg/mL diaminobenzidine, 100 μM cytochrome c, 2 μg/mL bovine catalase in 0.1 M PBS, pH 7.0). After staining, the sections were rinsed in PBS, dehydrated, and mounted. Three to five sections per each of the three hearts were analyzed with NIH ImageJ image analysis software (version 2.0.0-rc69/1.52p; Java 1.8.0_202, National Institutes of Health, Bethesda, MD, USA) as described [34,35].

### 2.6. Gene Expression and mtDNA Quantitation

Total RNA was isolated from ventricles using the innuSOLVE RNA Reagent (AnalytikJena, Gina, Germany) following the instructions of the manufacturer. cDNA was synthesized using the First Strand cDNA Synthesis Kit (Thermo Fischer Scientific, Waltham, MA, USA) using 1 μg of total RNA (treated with DNAse I (Thermo Fischer Scientific, Waltham, MA, USA)). Gene expression was normalized to the expression of the reference gene *Gapdh* (glyceraldehyde 3-phosphate dehydrogenase). Primers for qPCR were used for atrial natriuretic protein, *Nppa* (forward: 5′-CATCACCCTGGGCTTCTTCCT-3′; reverse: 5′-TGGGCTCCAATCCTGTCAATC-3′), brain natriuretic protein, *Nppb* (forward: 5′-GCGGCATGGATCTCCTGAAGG-3′; reverse: 5′-CCCAGGCAGAGTCAGAAACTG-3′3), *Myh7* (forward: 5′-ATGTGCCGGACCTTGGAA-3′; reverse: 5′-CCTCGGGTTAGCTGAGAGATCA-3′), *Myh6* (forward: 5′-GGCACAGAAACACCTGAAGA-3′; reverse: 5′-CATTGGCATGGACAGCATCATC-3′), *c-Myc* (forward: 5′-GCCCCTAGTGCTGCATGAG-3′; reverse: 5′-CCACAGACACCACATCAATTTCTT-3′), *c-Fos* (forward: 5′-CCGACTCCTTCTCCAGCAT-3′; reverse: 5′-TCACCGTGGGGATAAAGTTG-3′).

Total DNA was isolated both from the organic phase and interphase that remained after RNA precipitation according to the manufacturer’s instructions. Then, 10 ng of total DNA was used in qPCR. Two pairs of primers to different regions of mitochondrial genome were used: *mtND1* (forward: 5′-CTAGCAGAAACAAACCGGGC-3′; reverse: 5′-CCGGCTGCGTATTCTACGTT-3′) and *mt16S rDNA* (forward: 5′-CCGCAAGGGAAAGATGAAAGAC-3′; reverse: 5′-TCGTTTGGTTTCGGGGTTTC-3′). Data were normalized to nuclear hexokinase gene *nHK2* (forward: 5′-GCCAGCCTCTCCTGATTTTAGTGT-3′; reverse: 5′-GGGAACACAAAAGACCTCTTCTGG-3′). The n-Fold difference in gene expression and mtDNA quantity was calculated using 2^−ΔΔCT^ method (*n* = 5/group).

### 2.7. Western Blot

The left ventricles (LV) were homogenized in radioimmunoprecipitation assay buffer as described earlier [36]. Western blot was performed using the following antibodies: β-catenin (1:1000, sc-7963, Santa Cruz Biotechnology, Santa Cruz, CA, USA), Akt1 (1:1000, sc-1618, Santa Cruz Biotechnology), Lef-1 (1:1000, sc-374412, Santa Cruz Biotechnology, Santa Cruz, CA, USA), phosphorylated Akt at Ser^473^ (1:500, sc-101629, Santa Cruz Biotechnology), phosphorylated Akt at Thr^308^ (1:500, sc-135650, Santa Cruz Biotechnology, Santa Cruz, CA, USA), Erk1/2 (1:1000, 9102, Cell Signaling Technology, Danvers, MA, USA), phosphorylated Erk1/2 at Thr^202^/Thr^204^ (pErk1/2, 1:1000, 4377, Cell Signaling Technology, Danvers, MA, USA), AMPKα 1/2 (1:500, sc-25792, Santa Cruz Biotechnology), phosphorylated AMPKα at Thr^172^ (pAMPKα, 1:500, sc-33524-R, Santa Cruz Biotechnology), PKA (1:500, sc-390548, Santa Cruz Biotechnology), phosphorylated PKA (pPKA; 1:500, sc-32968, Santa Cruz Biotechnology), hormone-sensitive lipase (HSL; 1:1000, 4107, Cell Signaling Technology, Danvers, MA, USA), phosphorylated HSL (pHSL) at Ser^565^ (1:1000, 4137, Cell Signaling Technology, Danvers, MA, USA), pHSL at Ser^563^ (1:1000, 4139, Cell Signaling Technology, Danvers, MA, USA), acetyl-CoA carboxylase (ACC; 1:1000, 04-322, Millipore, Burlington, MA, USA), phosphorylated ACC (pACC; 1:500, 07-303, Millipore), Axin-1 (1:1000, 3323, Cell Signaling Technology, Danvers, MA, USA), α/β-hydrolase domain (ABHD5, 1:1000, sc-100468, Santa Cruz Biotechnology), adipose triglyceride lipase (ATGL, 1:1000, 2138, Cell Signaling Technology, Danvers, MA, USA), adenomatous polyposis coli (APC, 1:100, sc-896, Santa Cruz Biotechnology), carnitine palmitoyltransferase 1 (CPT1, 1:100, sc-31128, Santa Cruz Biotechnology), CD36 (1:100, sc-9154, Santa Cruz Biotechnology), G_0_S_2_ (1:100, sc-133423, Santa Cruz Biotechnology), GLUT-4 (1:100, sc-7938, Santa Cruz Biotechnology), pyruvate dehydrogenase kinase 1 (PDK1, 1:100, sc-7140, Santa Cruz Biotechnology), mammalian target of rapamycin (mTOR, 1:1000, 2983, Cell Signaling Technology, Danvers, MA, USA), phosphorylated at Ser^2448^ mTOR (1:1000, 5536, Cell Signaling), OXPHOS (1:1000, ab110413, Abcam, Cambridge, UK) β-actin (1:25,000, A3854, Sigma, St. Louis, MO, USA)and GAPDH (1:25,000, 5174, Cell Signaling) as a loading control. After exposure to horseradish peroxidase-conjugated secondary antibodies: Anti-mouse (1:15,000, sc-2005, Santa Cruz Biotechnology), anti-rabbit (1:10,000, sc-2004, Santa Cruz Biotechnology), and anti-goat (1:10,000, sc-2020, Santa Cruz Biotechnology) for one hour, target proteins were visualized by enhanced chemiluminescence (Pierce, Rockford, IL, USA). Proteins were quantified by densitometry (*n* = 5/group). 

### 2.8. Measurement of Lipids

Heart lipids were extracted from LV and measured as previously described [36]. Briefly, the lipids were separated by thin-layer chromatography on silica gel 60 plates (Merck, Fort Kenalworth, NJ, USA) in heptane/isopropyl ether/glacial acetic acid (60/40/4, *v*/*v*/*v*). To visualize lipid bands, the plate was soaked in the water mixture that contained 10% cupric sulphate and 8% phosphoric acid and then burned at 140 °C for 20 min. The lipids were quantified using densitometry (*n* = 5/group). 

### 2.9. Statistical Analysis

Differences between groups were evaluated using a nonparametric Kruskal–Wallis test followed by Dunn’s multiple-comparison post hoc test. The data are represented as mean ± standard deviation (SD). Values of *p* < 0.05 were considered statistically significant. The statistical analysis was performed using Prism software (GraphPad Prism, version 8.4.2, San Diego, CA, USA).

## 3. Results

### 3.1. Mice Heterozygous for β-Catenin Show Attenuated Heart Hypertrophy and Decreased Heart Rate

**Figure 1:**
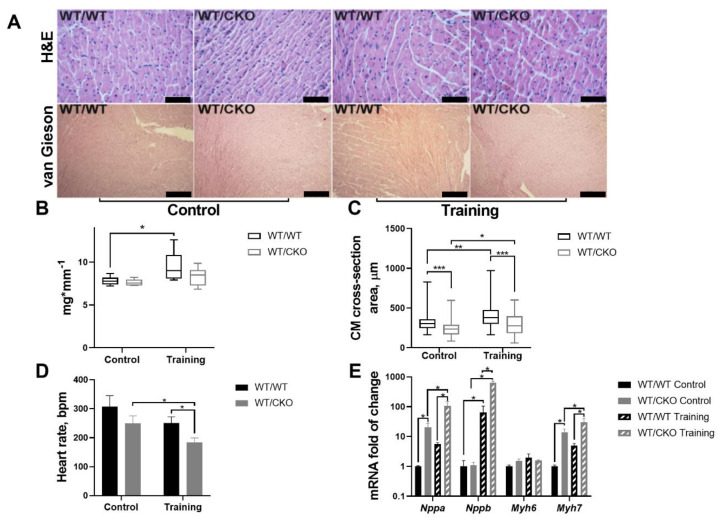
Heterozygous deletion of *β-catenin* does not affect adult heart morphology, but attenuates the hypertrophic response. (**A**) Top row— hematoxylin and eosin (H&E)-stained paraffin-embedded sections; 400× magnification, scale bar—50 μm. Bottom row—Picrofuchsin van Gieson-stained paraffin-embedded sections; 100× magnification, scale bar—200 μm for H&E and 100× for van Gieson; WT/WT—control; WT/CKO—heterozygous. (**B**) Heart weight/tibia length (HW/TL) ratio in trained and untrained mice. WT/WT control, *n* = 11, WT/WT training *n* = 11, WT/CKO control, *n* = 7, WT/CKO training *n* = 12. (**C**) Cardiomyocytes cross-section area in trained and untrained mice. Over 100 cells per heart from 3 hearts of each genotype were analyzed. (**D**) Electrophysiological monitoring of heart rate (beat/min) in trained and untrained mice. WT/WT control, *n* = 5, WT/WT training, *n* = 4, WT/CKO control, *n* = 4, WT/CKO training *n* = 4. (**E**) qPCR analysis of fetal gene expression in control and trained WT/WT and CKO/WT mice. *n* = 5. The data are expressed as the mean ± SD of arbitrary fold of change relative to control levels. * *p* < 0.05, ** *p* < 0.01, *** *p* < 0.005 (Kruskal–Wallis test followed by Dunn’s multiple-comparison post hoc test).

Hearts of WT/WT and WT/CKO mice displayed no histopathological abnormalities or fibrosis before or after training (Figure 1A). Training led to a significant increase in HW/TL in WT/WT, but there was no difference in the HW/TL ratio between WT/CKO trained and sedentary controls (*p* = 0.37) (Figure 1B). The cardiomyocytes’ cross-sectional area was significantly lower in the hearts of sedentary WT/CKO mice compared to matched WT/WT. Training led to an increase in cardiomyocytes width by 1.23 times in WT/WT mice and by 1.15 times in WT/CKO mice compared to the relevant sedentary mice. Of note, cardiomyocytes width in knockout mice that were heterozygous for *β-catenin* did not exceed this level in WT/WT mice after endurance training (Figure 1C).

We then tested the effect of heterozygous knockout of a *β-catenin* on heart rate and its ability to respond to endurance training. There were no signs of arrhythmia in sedentary and trained WT/CKO mice. Heart rate was decreased in WT/CKO mice compared to WT/WT animals in the absence of physiological stress (*p* = 0.062) (Figure 1D). Training similarly lowered the heart rate in *β-catenin* heterozygous and WT/WT mice after the six weeks of training while the heart rate of WT/CKO trained mice was significantly decreased (Figure 1D). 

Analysis of the expression of fetal genes (*Nppa*, *Nppb*, *Myh6*, and *Myh7*) in trained and untrained mice showed that *Nppa* and *Myh7* mRNA levels were significantly increased in hearts of sedentary WT/CKO mice compared to WT/WT (Figure 1E). Training induced expression of *Nppb* in the hearts of WT/WT and WT/CKO mice. Notably, the expression of *Nppb* in the hearts of trained WT/CKO mice was ten times higher than in trained WT/WT (Figure 1E). Expression of *Myh6* was upregulated only in the hearts of WT/WT mice after the training (Figure 1E). The expression of *Nppa* and *Myh7* were significantly upregulated only in the hearts of WT/CKO after the training compared to WT/WT trained mice (Figure 1E). 

Taken together, these results demonstrate that heterozygosity at *β-catenin* in the heart of adult untrained mice changes the pattern of the fetal gene expression, reduces the cardiomyocytes size, and decreases the heart rate. At the same time, *β-catenin* haploinsufficiency attenuates the hypertrophic response associated with upregulation of the fetal genes and causes more prominent reduction in heart rate after endurance training.

### 3.2. β-Catenin Haploinsufficiency Leads to Downregulation of Canonical Wnt Signaling in the Trained and Untrained Heart

**Figure 2:**
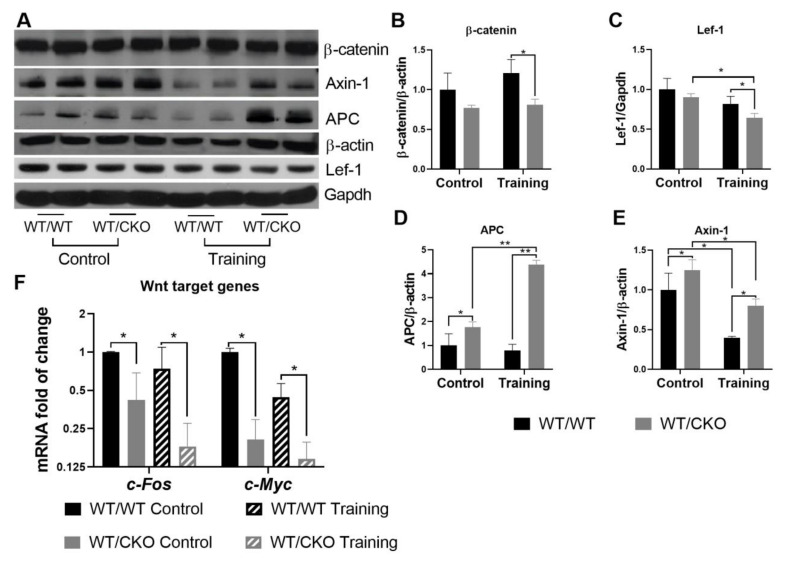
Heterozygous knockout of *β-catenin* leads to canonical Wnt signaling downregulation in the hearts of trained and untrained mice. (**A**) Western blot of total β-catenin, Lef-1, Axin-1, and adenomatous polyposis coli (APC) in left ventricle (LV) lysates from WT/WT and WT/CKO mice in sedentary conditions (Control) and after endurance training (Training). (**B**) Densitometry of total β-catenin normalized to total β-actin. (**C**) Densitometry of Lef-1 normalized to Gapdh. (**D**) Densitometry of Axin-1 normalized to β-actin. (**E**) Densitometry of APC normalized to β-actin. (**F**) qPCR analysis of β-catenin target gene expression. The data are expressed as the mean ± SD of arbitrary fold of change relative to control levels. *n* = 5/group. * *p* < 0.05, ** *p* < 0.01 (Kruskal–Wallis test followed by Dunn’s multiple-comparison post hoc test).

The levels of β-catenin and Lef-1 in total left ventricle lysates remained unchanged in the hearts of sedentary WT/CKO compared to WT/WT mice, which is consistent with previously published data [10] (Figure 2A–C). The protein levels of Axin-1 and APC were significantly higher in the hearts of untrained WT/CKO heterozygous mice compared to WT/WT mice (Figure 2A,D,E). The higher level of scaffold proteins suggests that β-catenin is retained in the cytoplasm. This observation is supported by a downregulation of *c-Myc* and *c-Fos* expression, both of which are β-catenin target genes (Figure 2F).

The levels of total β-catenin and nuclear β-catenin transcriptional co-activator Lef-1 were significantly lower in the hearts of WT/CKO mice after the training than in the hearts of WT/WT trained mice (Figure 2A–C). Training stimulated APC protein expression only in WT/CKO mice, while the level of Axin-1 in WT/WT and WT/CKO trained groups was reduced compared to their sedentary controls (Figure 2A,D,E). The levels of APC and Axin-1 were significantly higher in trained WT/CKO mice compared to WT/WT counterparts. These data suggest that β-catenin transcriptional activity in the hearts of trained WT/CKO mice decreases, which is consistent with the lower expression of β-catenin target genes (*c-Myc* and *c-Fos*; Figure 2F) in the hearts of WT/CKO mice after the training. 

### 3.3. Heterozygous Cardiospecific Knockout of β-Catenin Affects Hypertrophic Signaling in the Adult Heart

**Figure 3:**
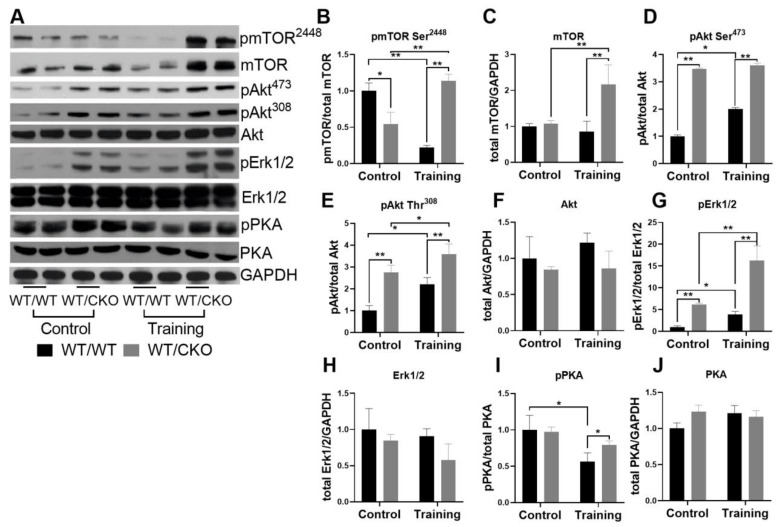
Heterozygous knockout of *β-catenin* affects hypertrophic signaling in the heart. (**A**) Western blot of pmTOR^2448^, mTOR, pAkt at Thr^308^, pAkt at Ser^473^, Akt, pErk1/2, Erk1/2, phosphorylated PKA (pPKA) and protein kinase A (PKA) in LV lysates from WT/WT and WT/CKO mice in sedentary conditions (Control) and after the endurance training (Training). (**B**) Densitometry of pmTOR at Ser^2448^ normalized to mTOR. (**C**) Densitometry of total mTOR normalized to GAPDH. (**D**) Densitometry of pAkt at Ser^473^ normalized to Akt. (**E**) Densitometry of pAkt at Thr^308^ normalized to Akt. (**F**) Densitometry of total Akt normalized to GAPDH. (**G**) Densitometry of pErk1/2 normalized to total Erk1/2. (**H**) Densitometry of total Erk1/2 normalized to GAPDH. (**I**) Densitometry of pPKA normalized to total PKA. (**J**) Densitometry of total PKA normalized to GAPDH. The data are expressed as the mean ± SD of arbitrary fold of change relative to control levels. *n* = 5/group. * *p* < 0.05, ** *p* < 0.01 (Kruskal-Wallis test followed by Dunn’s multiple-comparison post hoc test).

Considering the complex regulation of cardiac metabolism, we tested the activity of Pi3K/Akt, cAMP/PKA, and MEK1-Erk1/2 signaling pathways. The level of mechanistic target of rapamycin (mTOR) phosphorylation at Ser^2448^ in the hearts of sedentary WT/CKO mice was significantly lower compared to untrained WT/WT mice (Figure 3A,B), and there were no changes in the level of total mTOR (Figure 3A,C). The level of Akt phosphorylation at Thr^308^ and Ser^473^ (Figure 3A,D,E) was significantly higher in the hearts of untrained WT/CKO mice compared to WT/WT untrained controls. We did not observe differences in the level of total Akt in any groups of animals (Figure 3A,F). The level of pErk1/2 was more than five times higher in the hearts of untrained WT/CKO compared to untrained WT/WT mice, and the level of total Erk1/2 was the same (Figure 3A,G,H). The levels of pPKA and total PKA were unchanged in the hearts of untrained WT/CKO mice compared to sedentary WT/WT mice (Figure 3A,I,J).

Endurance training decreased the level of pmTOR in WT/WT mice, but increased it in WT/CKO mice compared to sedentary mice of the same genotype (Figure 3A,B). Moreover, the level of pmTOR was much higher in trained mutant mice compared to trained WT/WT mice (Figure 3A,B). Training significantly increased the level of total mTOR only in WT/CKO mice (Figure 3A,C). The levels of phosphorylation of Akt at Ser^473^ (Figure 3A,D) and at Thr^308^ (Figure 3A,E) were twice as high in the hearts of trained WT/WT mice, but only slightly increased in WT/CKO mice compared to untrained control mice with the same genotype. The level of pAkt in trained WT/WT mice did not reach the level of pAkt in trained WT/CKO mice (Figure 3A,D,E).

Endurance training resulted in the increased level of activative Erk1/2 in the hearts of mice of both genotypes (Figure 3A,G), which is indicative of the adaptation of the heart to stress. At the same time, the level of pErk1/2 was more than five-fold higher in the hearts of WT/CKO trained mice than in WT/WT trained mice (Figure 3A,G). The levels of pPKA in WT/WT mice after endurance training were significantly lower than in the relevant controls (Figure 3A,I). However, there was a significantly higher level of pPKA in the hearts of trained WT/CKO mice compared to trained WT/WT animals (Figure 3A,I). 

### 3.4. Heterozygous Ablation of β-Catenin Leads to Perturbation of Cardiac Lipid Metabolism

**Figure 4:**
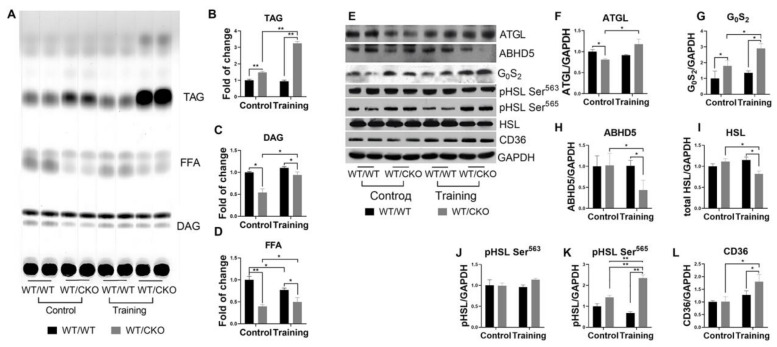
Heterozygous deletion of the *β-catenin* leads to the inhibition of triacylglycerols (TAG) hydrolysis in the hearts of trained and untrained WT/CKO mice. (**A**) Thin layer chromatography of lipids extracted from LV from WT/WT and WT/CKO mice in sedentary conditions (Control) and after the endurance training (Training). (**B**) Densitometry of TAG. (**C**) Densitometry of diacylglycerols (DAG). (**D**) Densitometry of free fatty acids (FFA). (**E**) Western blot of ATGL, ABHD5, G_0_S_2_, pHSL at Ser^563^, pHSL at Ser^565^, total HSL, and in LV lysates from WT/WT and WT/CKO mice in sedentary conditions (Control) and after the endurance training (Training). (**F**) Densitometry of ATGL protein normalized to GAPDH. (**G**) Densitometry of G_0_S_2_ protein normalized to GAPDH. (**H**) Densitometry of ABHD5 protein normalized to Gapdh; (**I**) Densitometry of HSL protein normalized to GAPDH. (**J**) Densitometry of pHSL at Ser^563^ normalized to total HSL. (**K**) Densitometry of pHSL at Ser^565^ normalized to total HSL. (**L**) Densitometry of CD36 protein normalized to GAPDH. The data are expressed as the mean ± SD of arbitrary fold of change relative to control levels. *n* = 5/group. * *p* < 0.05, ** *p* < 0.01 (Kruskal–Wallis test followed by Dunn’s multiple-comparison post hoc test).

Hearts of sedentary WT/CKO mice showed an increase in TAG content, but a decrease in diacylglycerols (DAG) and free fatty acids (FFA) compared to untrained WT/WT controls (Figure 4A–D). We did not observe differences in TAG and DAG content in the hearts of trained WT/WT mice compared to untrained mice with the same genotype (Figure 4A–C). Only FFA levels were lower in the hearts of trained WT/WT mice (Figure 4A,D). Training stimulates the accumulation of TAG in hearts of WT/CKO mice compared with sedentary WT/CKO mice (Figure 4A,B). The DAG and FFA levels increased in WT/CKO hearts after the training compared to untrained mice (Figure 4A,B) but still were significantly lower in the hearts of trained heterozygous animals compared to wild type trained controls (Figure 4A–C). 

The protein level of adipose tissue triacylglycerol lipase (ATGL), an enzyme which catalyzes the hydrolysis of TAG, was significantly lower in untrained WT/CKO mice compared to relevant WT/WT mice. Additionally, the level of ATGL inhibitor G_0_S_2_ was increased in sedentary WT/CKO mice, whereas the level of α/β-hydrolase domain containing 5 (ABHD5) protein, its activator, was unchanged (Figure 4E–H). The analysis of total and phosphorylated hormone sensitive lipase (HSL) levels also indicated the inhibition of TAG and DAG hydrolysis in untrained WT/CKO compared to relevant WT/WT mice. The levels of total and phosphorylated HSL at Ser^563^, hence active, were unchanged in untrained WT/CKO compared to WT/WT mice (Figure 4E,I,J). The phosphorylation level of HSL at Ser^565^, hence inhibited, was higher in the hearts of WT/CKO untrained mice compared to untrained WT/WT mice (Figure 4E,K). Endurance training did not affect the protein levels of ATGL, G_0_S_2_, and ABHD5 in the hearts of WT/WT mice (Figure 4E–H). In the hearts of WT/CKO mice following endurance training, the level of ATGL increased slightly, while G_0_S_2_, a regulator of lipolysis, increased by 50%. Concomitantly, the level of ABHD5, an ATGL co-activator, decreased (Figure 4E–H). The protein level of G_0_S_2_ was higher, and the level of ABHD5 was lower in trained WT/CKO compared to trained WT/WT mice (Figure 4E–H). The levels of phosphorylated HSL at Ser^565^ and at Ser^563^ were unchanged in the hearts of WT/WT mice after the training (Figure 4E,I–K). The level of total HSL was reduced in trained WT/CKO mice compared to their untrained counterparts (Figure 4E,I). The phosphorylation level of HSL at Ser^565^ increased two-fold in trained WT/CKO compared to their untrained counterparts, while the phosphorylation level of HSL at Ser^563^ was unchanged (Figure 4E,I–K). Thus, the level of total HSL was lower, whereas the level of inhibited HSL (phosphorylated at Ser^565^) was higher in trained WT/CKO compared to trained WT/WT mice (Figure 4E,I–K). Analyses of protein involved in the transport of FA across the membrane revealed that endurance training might activate these processes in the cardiomyocytes of WT/CKO mice. The level of CD36 was higher in the hearts of WT/CKO trained mice compared to WT/WT controls (Figure 4E,L).

**Figure 5:**
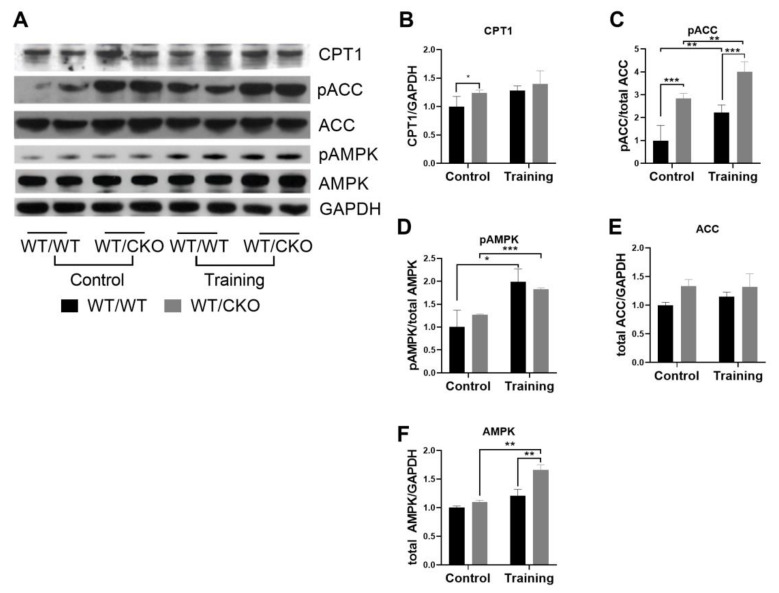
Heterozygous knockout of β-catenin alters the fatty acids (FA) β-oxidation in hearts of trained and untrained WT/CKO mice. (**A**) Western blot of CPT1, pACC at Ser79, acetyl-CoA carboxylase (ACC), pAMPK at Thr172, total AMP-activated protein kinase (AMPK) in LV lysates from WT/WT and WT/CKO mice in sedentary conditions (Control) and after the endurance training (Training). (**B**) Densitometry of CPT1 protein normalized to GAPDH. (**C**) Densitometry of pACC at Ser79 normalized to total ACC. (**D**) Densitometry of pAMPK at Thr172 normalized to total AMPK. (**E**) Densitometry of total ACC normalized to GAPDH. (**F**) Densitometry of total AMPK normalized to GAPDH. The data are expressed as the mean ± SD of arbitrary fold of change relative to control levels. *n* = 5/group. * *p* < 0.05, ** *p* < 0.01, *** *p* < 0.005 (Kruskal–Wallis test followed by Dunn’s multiple-comparison post hoc test).

The FA β-oxidation is crucial for heart function and is controlled by many factors. A rate-limiting step of β-oxidation is the transport of FA through the mitochondrial membrane, which is catalyzed by CPT1. Malonyl-CoA is the product of acetyl-CoA carboxylase (ACC) and is the allosteric inhibitor of carnitine palmitoyltransferase I (CPT1) activity. The protein level of CPT1 was higher in the hearts of sedentary WT/CKO compared with WT/WT, but it was unchanged in the hearts of WT/WT and WT/CKO after the exercise (Figure 5A,B). The level of inhibited pACC was three times higher in the hearts of untrained WT/CKO mice compared to matched WT/WT mice (Figure 5A,C). While training induced phosphorylation of ACC in WT/WT and WT/CKO mice, phosphorylation of WT/CKO was two times higher in WT/CKO mice. The level of total ACC remained unchanged (Figure 5A,E). Notably, there was no difference in the level of stimulatory phosphorylation of AMPK at Thr172 in control WT/WT and WT/CKO mice, while AMPK phosphorylation at Thr172 increased in both groups of mice after the training (Figure 5A,D). Interestingly, the level of total AMPK was enhanced by the endurance training only in the WT/CKO group (Figure 5A,F).

### 3.5. β-Catenin Heterozygosity Affects the Level of Proteins Involved in Glucose Metabolism

**Figure 6:**
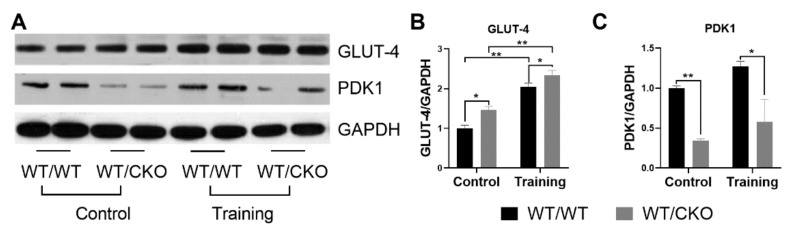
Heterozygous deletion of *β-catenin* activates glucose metabolism in the heart. (**A**) Western blot of total GLUT-4, PDK1 in LV lysates from WT/WT and WT/CKO mice in sedentary conditions (Control) and after the endurance training (Training). (**B**) Densitometry of GLUT-4 protein normalized to GAPDH. (**C**) Densitometry of PDK1 protein normalized to GAPDH. The data are expressed as the mean ± SD of arbitrary fold of change relative to control levels. *n* = 5/group. * *p* < 0.05, ** *p* < 0.01 (Kruskal-Wallis test followed by Dunn’s multiple-comparison post hoc test).

The protein level of GLUT-4, the most abundant glucose transporter in the heart, was significantly higher in hearts of sedentary WT/CKO mice compared to their matched WT/WT control (Figure 6A,B). Training stimulated GLUT-4 protein expression in both WT/WT and WT/CKO mice, which is noticeable for the adaptation of the heart to training (Figure 6A,B). In the hearts of trained WT/CKO mice, the level of GLUT-4 was higher compared to WT/WT mice. Additionally, the level of pyruvate dehydrogenase kinase 1 (PDK1) was significantly lower in the hearts of WT/CKO compared to WT/WT mice in both trained and untrained groups (Figure 6A,C). These results might suggest the activation of glucose metabolism in WT/CKO cardiomyocytes.

### 3.6. Heterozygous Cardiospecific Knockout of β-Catenin Reduces the Number of Mitochondria and Impairs Their Function in Hearts of Trained Mice

**Figure 7:**
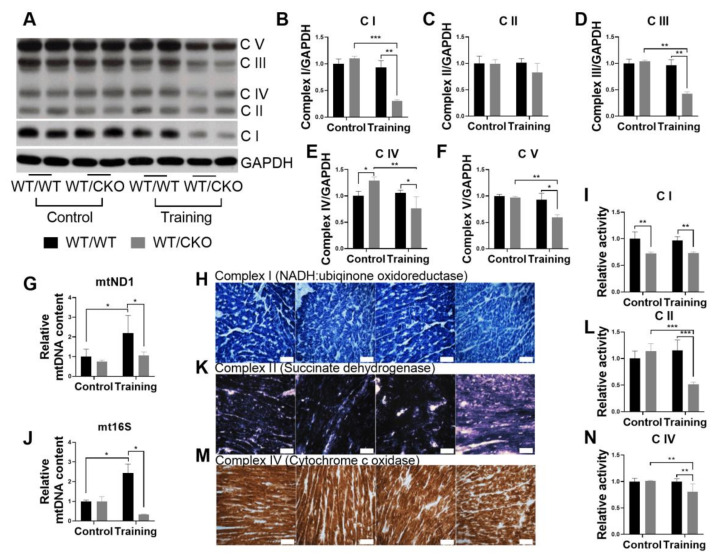
Heterozygous knockout of β-catenin decreases the level of mtDNA and inhibits oxidative phosphorylation in hearts of trained mice. (**A**) Western blot of total complex I (C I), complex II (C II), complex III (C III), complex IV (C IV), and complex V (C V) in LV lysates from WT/WT and WT/CKO mice in sedentary conditions (Control) and after endurance training (Training). (**B**) Densitometry of Complex I normalized to GAPDH. (**C**) Densitometry of Complex II normalized to GAPDH. (**D**) Densitometry of Complex III normalized to GAPDH. (**E**) Densitometry of Complex IV normalized to GAPDH. (**F**) Densitometry of Complex V normalized to GAPDH. (**G**,**J**) qPCR analysis of mtDNA expression normalized to nuclear hexokinase gene. (**H**,**K**,**M**) Histochemical evaluation of Complex I, II, and IV activity on cryosections of hearts from WT/WT and WT/CKO mice. Scale bar—50 μm. (**I**) Relative activity NADH-coenzyme Q oxidoreductase (Complex I). (**L**) Relative activity of succinate dehydrogenase (Complex II). (**N**) Relative activity of cytochrome c oxidase (Complex IV). The data are expressed as the mean ± SD of arbitrary fold of change relative to control levels. *n* = 5/group for western blot and mtDNA quantification and *n* = 3/group for histochemistry. * *p* < 0.05, ** *p* < 0.01, *** *p* < 0.005 (Kruskal-Wallis test followed by Dunn’s multiple-comparison post hoc test).

To quantify the number of mitochondrial genomes, as the surrogate marker of the number of mitochondria present in the cardiomyocytes of two genotypes under resting or trained conditions, we performed qPCR for two different regions of the mitochondrial genome: mtND1 and mt16S rDNA (Figure 7G,J). The levels of mitochondrial DNA (mtDNA) and OXPHOS proteins (except for complex IV, which was higher in WT/CKO), as well as the activity of OXPHOS complexes II and IV, were similar between untrained WT/CKO and WT/WT mice, while the activity of complex I was decreased (Figure 7A–F,H,I,K–N). The level of mtDNA increased in the hearts of WT/WT mice after the training, indicative of athletic heart development, while training did not increase the mtDNA content in hearts of WT/CKO mice (Figure 7G,J). The level of mtDNA in myocardium of WT/CKO mice was significantly lower than that of trained WT/WT mice (Figure 7G,J). Training did not affect the levels or activity of OXPHOS complexes in WT/WT mice, but it decreased OXPHOS protein content and inhibited their activities in WT/CKO cardiomyocytes. The levels of almost all OXPHOS subunits were lower in the hearts of trained WT/CKO than in the hearts of trained WT/WT control mice (Figure 7A,B,D–F). Similarly, the activities of complexes I, II, and IV were lower in trained WT/CKO compared to trained WT/WT mice (Figure 7H,I,K–N). Overall, these data show that the mtDNA level is decreased and oxidative phosphorylation is inhibited in the hearts of trained WT/CKO mice compared with wild type controls.

## 4. Discussion

**Figure 8:**
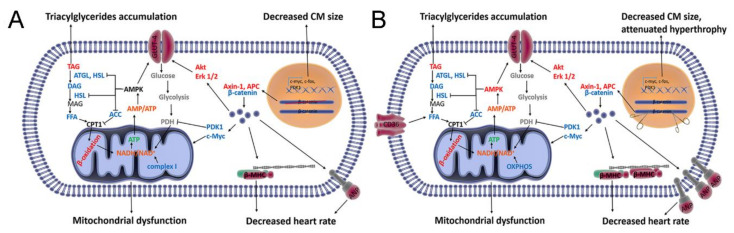
Schematic representation of the canonical Wnt signaling function in regulation of cardiac metabolism under the sedentary conditions (**A**) and after the endurance training (**B**). (**A**) Heterozygous knockout of *β-catenin* inhibits canonical Wnt signaling and downregulates β-catenin target genes (*c-Myc*, *c-Fos*). This leads to decreased cardiomyocytes size. The higher levels of ANP and β-MHC may lead to the lower heart rate. Canonical Wnt signaling is involved in FA metabolism and regulation of mitochondria function via its targets c-Myc and PDK1. Decreased canonical Wnt signaling is associated with the activation of Pi3K–Akt and MAPK/Erk1/2 signaling pathways. Altogether, this causes the inhibition of lipolysis and the activation of glucose uptake in hearts of WT/CKO mice. Activation of β-oxidation and glucose oxidation along with lower activity of complex I lead to the accumulation of NADH, which promotes mitochondrial dysfunction. (**B**) The lower level of canonical Wnt signaling attenuates the cardiomyocytes hypertrophy. Adaptation of WT/CKO mice to the endurance training is accompanied by activation of AMPK and a stronger activation of pre-activated Pi3K–AKT and MAPK/Erk1/2 signaling pathways. Increased AMPK leads to the inhibition of lipolysis and activation of β-oxidation. Activation of AMPK, Pi3K–AKT, and MAPK/Erk1/2 signaling pathways stimulates FA and glucose uptake. Downregulation of canonical Wnt signaling reduces mitochondria biogenesis and OXPHOS activity in the heart during adaptation to the endurance training. Decreased OXPHOS protein level and activity along with enhanced β-oxidation further exacerbate mitochondrial dysfunction. Notes: Red—observed increase; blue—observed decrease; orange—possible increase; green—possible decrease; black—remain unchanged relative to relevant WT/WT mice.

The key role of canonical Wnt signaling in maladaptive heart remodeling is well established, since the activation of β-catenin is necessary for pathological heart remodeling, which is associated with cardiomyocytes hypertrophy [9,10,11,12,13,14,15]. However, the function of β-catenin signaling in the development of physiological cardiac hypertrophy induced by exercise training is unknown. Moreover, despite the intense research of the β-catenin role in cellular metabolism, its function in the adult heart still remains largely unknown. Our data show that β-catenin plays an essential role in regulating postnatal cardiac metabolism and sustains the homeostatic balance of signaling networks under physiological conditions and during the adaptation to training (Figure 8). Our findings demonstrate that Wnt/β-catenin signaling not only regulates maladaptive heart remodeling [9,10], but also physiological heart adaptation to training through the maintenance of metabolic balance in the heart.

In the present study, the reduction in Wnt/β-catenin signaling activity is supported by the increased levels of the key components of the β-catenin degradation complex (Axin-1 and APC) and a decreased level of its transcriptional co-activator Lef-1, along with downregulation of β-catenin targets (i.e., *c-Myc*, *c-Fos* and PDK1). The downregulation of Wnt/β-catenin activity causes a decrease in cardiomyocytes width and leads to upregulation of fetal genes (*Nppa*, *Nppb,* and *Myh7*) (Figure 8A). This observation is consistent with published data showing that cardiomyocytes growth is restricted after the inducible knockout of *β-catenin* [9]. On the contrary, stabilization of β-catenin leads to cardiomyocytes hypertrophy under normal conditions and pathological conditions through Lef-1 [6,37]. 

Heart rate reflects cardiac function and the ability of an organism to respond to many types of stress, including endurance training. The lower heart rate we observe in sedentary WT/CKO mice might have been caused by upregulation of *Nppa*, which encodes ANP and has a bradycardiac effect [38,39]. Furthermore, consistent with our results, mice that overexpress *Myh7* (encodes β-MHC) have a depressed cardiac contractility [40,41], which may exert an additional impact on the reduced heart rate we see in WT/CKO mice (Figure 8A). 

Due to the crosstalk between the Wnt/β-catenin, Pi3K–Akt, and MAPK/Erk1/2 signaling pathways, inhibition of any one signaling cascade can be compensated by activation of another [8]. We observed the activation of Erk1/2 and Akt in the hearts of sedentary WT/CKO mice, the key signal transducers required for the developmental growth of cardiomyocytes. However, the activation of Erk1/2 and Akt appears to be insufficient to compensate for the deficiency of β-catenin transcriptional function, since a reduced cardiomyocytes width is observed in WT/CKO mice.

Circulating FFA and hydrolysis of endogenous TAG are the major sources of FA for β-oxidation in heart muscle [42]. We observed an inhibition of lipolysis in sedentary WT/CKO mice, as evidenced by the accumulation of TAG and decrease in FFA content. These changes, in turn, might be due to an inhibition of ATGL and HSL activities. The next step of FA catabolism is performed by mitochondria through β-oxidation. ACC is a key regulator of this process, and its inhibition leads to enhanced β-oxidation [43]. Sedentary WT/CKO mice exhibit a much higher level of phosphorylated and, hence, inhibited ACC, indicative of an activation of mitochondrial FA transport (mediated by CPT1) and further β-oxidation (Figure 8A). The higher level of GLUT-4 we observe in WT/CKO hearts may provide a compensatory energy source, consistent with a previous report that glucose uptake is activated in the heart following *ATGL* knockout [44]. A reduced level of the direct β-catenin target PDK1 is indicative of a loss of inhibitory phosphorylation of pyruvate dehydrogenase complex [45] in the hearts of sedentary WT/CKO mice. This might suggest an activation of oxidative glucose metabolism followed by enhanced respiration (Figure 8A). Surprisingly, the WT/CKO mice have a lower activity of OXPHOS complex I, which indicates the perturbation of mitochondrial NADH oxidization. As was modeled and experimentally validated, a deficiency in complex I induces a compensatory activation of β-oxidation, which impairs OXPHOS function and exacerbates mitochondrial dysfunction [46,47], which is in line with our results (Figure 8A). Of importance, the reduced activity of complex I also increase the NADH/NAD+ ratio, which may inhibit activity of cellular deacetylases, especially Sirt3, which leads to mitochondrial pore opening and exacerbates mitochondrial dysfunction. [47,48].

As becomes clear from our study, Wnt/β-catenin signaling activity is indispensable for heart adaptation to endurance training. Accordingly, the activity of canonical Wnt signaling in the hearts of WT/CKO mice is lower after the training compared to WT/WT animals (Figure 8B). Moreover, the hypertrophic cardiomyocytes growth is attenuated in WT/CKO mice after the endurance training, although the hypertrophic genes are upregulated (i.e., *Nppa*, *Nppb,* and *Myh6*). Endurance training slows down heart rate in both groups of mice, but the decline is more prominent in trained WT/CKO mice. Additionally, training stimulates the expression of *Nppa* and *Myh7* only in WT/CKO mice, and this can further contribute to the development of bradycardia. Therefore, our results demonstrate that Wnt/β-catenin signaling is required not only for pathological heart remodeling [9,10], but also for training-induced cardiomyocytes hypertrophy (Figure 8B). Although, the limitations of this study are that we used only male mice, while it is known that there is a sex difference in response to exercises. Further studies will be needed to analyze the involvement of β-catenin in training-induced heart adaptation in female mice.

Heart remodeling is a complex process, involving the changes at the molecular, biochemical, and morphological levels, which are orchestrated by multiple signaling pathways, including Pi3K–Akt, PKA, and MAPK/Erk1/2 [49,50,51,52]. Not surprisingly, adaptation to training is accompanied by activation of Pi3K–Akt and MAPK/Erk1/2 signaling cascades both in WT/WT and WT/CKO mice (Figure 8B). Intriguingly, *β-catenin* haploinsufficiency leads to a stronger activation of Akt and Erk1/2 after the training. Adaptation to training is also accompanied by activation of AMPK, which is a key regulator of energy metabolism and is involved in metabolic plasticity during hypertrophic response [51]. There is a similar increase in the level of phosphorylated AMPK in both trained groups of mice compared with their sedentary counterparts. However, the level of total AMPK is significantly higher in the trained WT/CKO mice compared to WT/WT. The latter explains the enhanced phosphorylation of ACC at Ser^79^ (suggests activation of β-oxidation) and phosphorylation of HSL at Ser^565^ (suggests reduced lipolysis) in the hearts of trained WT/CKO mice (Figure 8B). 

GLUT-4 and CD36 are the predominant transporters for glucose and FA in the heart, respectively. Heart adaptation to exercise is accompanied by increased expression of both transporters, resulting in the activation of glucose and FA uptake [52,53]. Accordingly, there are significantly higher levels of GLUT-4 and CD36 in WT/CKO trained mice compared to those in WT/WT mice (Figure 8B). The mechanism underlying this may be related to the activation of AMPK during the adaptation to training in both groups of mice. Given the higher levels of active Akt and Erk1/2 in the hearts of trained WT/CKO mice, induced protein expression of both transporters is greater in mutant trained mice compared to trained WT/WT animals. 

Canonical Wnt signaling plays a well-established role in mitochondria homeostasis [19,20,21]. We found that the level of mtDNA in the hearts of WT/CKO mice remains unchanged following exercise. This is in contrast to WT/WT mice, in which the level of mtDNA increases after training. Furthermore, we suggest that the primary dysfunction of OXPHOS complex I along with increased level of CD36 (indicative of enhanced FA uptake) and phosphorylation of ACC at Ser^79^ (indicative of activation of β-oxidation) in trained WT/CKO mice, thus exhausting OXPHOS function and further exacerbating mitochondrial dysfunction [48]. Indeed, the levels of key subunits of OXPHOS complexes I, III, IV, and V in the hearts of WT/CKO mice decrease after the training (Figure 8B). In aggregate, these findings indicate that β-catenin ensures mitochondrial health and maintains mitochondrial activity during the adaptation of the heart to the endurance training. As it was previously reported, β-catenin promotes mitochondrial function through c-Myc, which is a pivotal mediator of mitochondrial biogenesis, activity of OXPHOS, and the citric acid cycle [20,54,55]. We suggest that the downregulation of c-Myc in the *β-catenin* haploinsufficient heart may be responsible for the impaired mitochondrial function during the training.

## Abbreviations

ABHD5	α/β-hydrolase domain containing 5
ACC	acetyl-CoA carboxylase
AMPK	AMP-activated protein kinase
ATGL	adipose tissue triacylglycerol lipase
CPT1	carnitine palmitoyltransferase I
DAG	Diacylglycerols
ECG	Electrocardiogram
FA	fatty acids
FFA	free fatty acids
HSL	hormone sensitive lipase
MAPK/Erk1/2	mitogen-activated protein kinase/extracellular signal-regulated kinases ½
mtDNA	mitochondrial DNA
mTOR	mechanistic target of rapamycin
OXPHOS	oxidative phosphorylation
PDK1	pyruvate dehydrogenase kinase 1
Pi3K	phosphoinositide-3-kinase
PKA	cyclic adenosine monophosphate-dependent protein kinase
PPARα	peroxisome proliferator-activated receptor α
TAG	triacylglycerols
